# TGF-β1 upregulates Sar1a expression and induces procollagen-I secretion in hypertrophic scarring fibroblasts

**DOI:** 10.1515/med-2022-0543

**Published:** 2022-09-17

**Authors:** Keun Jae Ahn, Jun-Sub Kim

**Affiliations:** Department of Science Education, Jeju National University, Jeju, 63063, Korea; Department of Biotechnology, Korea National University of Transportation, Chungbuk, 27909, Korea

**Keywords:** TGF-β1, Sar1a, COPII vesicle, procollagen secretion, hypertrophic scarring fibroblasts

## Abstract

Hypertrophic scarring (HTS) is a common fibroproliferative disorder that typically follows thermal and other injuries involving the deep dermis. The underlying pathogenic mechanisms are regulated by transforming growth factor-β (TGF-β); however, the exact mechanisms in HTS have not been elucidated. We conducted this study to explore the cellular signaling mechanisms for expression of Sar1a, a coat protein complex II-associated small GTPase, in HTS fibroblasts (HTSF). We found that Sar1a was upregulated in HTSF as compared to that in normal fibroblasts. Furthermore, stimulation of TGF-β1 increased the expression of Sar1a in HTSF, and small interfering RNA for Sar1a suppressed procollagen-I (PC-I) secretion. Next we investigated the signaling mechanism from TGF-β1 to Sar1a expression and its association with PC-I secretion. In the presence of TGF-β-activated kinase 1 (TAK1), c-Jun N-terminal kinase, or p38 inhibitors, the effect of TGF-β1 on Sar1a expression and PC-I secretion significantly decreased; however, it had no effect on collagen-1A (Col-1A) expression. Further, the inhibitors of Smad3 or extracellular signal-regulated kinases inhibited TGF-β1-induced Col-1A expression but had no effect on PC-I secretion and Sar1a expression. Taken together, our results suggested that TGF-β1 induces Sar1a expression through TAK1 signaling and this signaling event regulates PC-I secretion in HTSF.

## 1 Introduction

Hypertrophic scarring (HTS) is a common fibroproliferative disorder of the human dermis characterized by erythematous, raised, pruritic lesions of healing skin, which usually follows thermal and other injuries that involve the deep dermis [1]. Some hypertrophic scars, particularly those associated with thermal injuries, are associated with contractures [2,3]. During wound healing for tissue remodeling, complicated interactions take place within a complex network of profibrotic and antifibrotic molecules such as growth factors, proteolytic enzymes, and extracellular matrix (ECM) proteins [4].

Transforming growth factor-β1 (TGF-β1) contributes to wound healing via stimulation of angiogenesis, proliferation of fibroblasts, differentiation of myofibroblasts, synthesis of collagen (Col), and deposition of ECM proteins [5,6]. In burn patients with HTS, serum TGF-β1 level is upregulated locally and systemically [7]. The abnormal intracellular signaling of TGF-β1 is thought to initiate HTS by inducing the fibroblasts to excessively synthesize ECM and regulate connective tissue growth factor (CTGF), a downstream mediator of TGF-β1 [8,9,10]. As the main component of TGF-β1 signaling pathway, activation of Smad proteins leads to an increased expression of Col-1, -3, and -4 [11]. Although, non-Smad signaling pathways such as the mitogen-activated protein kinase (MAPK), extracellular signal-regulated kinase (ERK), and c-Jun N-terminal kinase (JNK) pathways have been associated with TGF-β signaling [12,13], their exact mechanisms in HTS have not been elucidated.

Collagen is essential for cell-cell interactions and cell attachment to the basement membrane. It is indispensable for skin formation, organization of cells into tissues, and tissue function [14]. Especially, myofibroblasts actively express Col and mediate fibrogenesis [15]. The expression levels of Col-1 or -3 are higher in HTS fibroblasts (HTSF) than in normal fibroblasts (NF) [10,16,17]. Excessive expression of Col is secreted and accumulated in pulmonary fibrosis, cirrhosis, cardiovascular diseases, and scars [15].

Type 1 Col is encoded by two genes, Col-1A1 and Col-1A2, their transcription rates are tightly coordinated [18]. Their promoter has various transcription factors binding sites such as specificity protein 1 (Sp1), Sp3, CCAAT-binding factor (CBF), collagen-Kruppel box, nuclear factor 1, activating protein-1 (AP-1), and AP-2 [19,20,21]. Although most studies have demonstrated that the transcriptional activation of Col-1A1 and Col-1A2 by TGF-β is regulated by Smad-dependent signaling [22], a clear understanding of the transcriptional mechanisms is still lacking.

Translated peptides from Col mRNA are transported into the endoplasmic reticulum (ER) to form triple helix procollagen (PC). The newly formed trimer is packaged in a coat protein complex II (COPII) cage in the ER. However, normal COPII cages are typically less than 90 nm in diameter, making it difficult to maintain trimerized Col up to 300 nm in cage [23]. Trimerized Col is associated with the TANGO1 complex responsible for the assembly of bulky COPII cages and the loading of Col into the cage from the ER exit site [24]. Collagen is delivered to the Golgi apparatus for final transformation. The end product is secreted out of the cells of the secretory granule through a plasma membrane protrusion [14].

In the present study, we analyzed the alteration of Sar1a, a protein involved in the formation of COPII vesicles between NF and HTSF. Furthermore, we explored the role of Sar1a expression in PC-I secretion and cellular signaling mechanisms for Sar1a expression in HTSF.

## 2 Materials and methods

### 2.1 Primary cell culture

Human skin biopsies were obtained from the tissue biobank of Hangang Sacred Heart Hospital. NFs used in this study were derived from skin biopsy, while HTSFs were isolated from burn-injured HTS tissues derived from surgical procedures, and the NFs and HTSFs were matched from four patients. The scars ranged in age from 1 to 2 years. The study was approved by the National University of Transportation Institutional Review Board (KNUT IRB 2022-17). Briefly, skin and scar tissues were cut into small pieces, soaked in dispase II (Gibco, Waltham, MA, USA) solution, and maintained at 4°C overnight. The next day, the epidermis was separated from the dermis, and the dermis was digested with collagenase type IV solution (500 U/mL) at 37°C for 30 min (Gibco, Waltham, MA, USA). The samples were inactivated with complete medium (DMEM) containing 10% fetal bovine serum (FBS) and 1% antibiotic-antimycotic containing penicillin, streptomycin, and amphotericin B (Gibco, Waltham, MA, USA), filtered, and centrifuged at 300×*g* for 5 min. The pellet was resuspended in complete medium, followed by culture at 37°C in 5% CO_2_. HTSFs at passage 2 were used for all the experiments

### 2.2 Reagents

Recombinant TGF-β1, TGF-β inhibitor (LY2109761), selective Smad3 inhibitor (SIS3), TGF-β activated kinase 1 (TAK1) inhibitor (EDHS-206), JNK inhibitor (SP600125), ERK inhibitor (PD98059), and p38 inhibitor (SB203580) were purchased from Sigma-Aldrich (St. Louis, MO, USA). FITC-conjugated secondary antibody and diamidino-2-phenylindole solution were purchased from Invitrogen (Carlsbad, CA, USA). Antibodies against CTGF, Sar1a, Sar1b, Sec13, Sec31a (Santa Cruz, Dallas, TX, USA), Sec23a, Sec24a (Abcam, Cambridge, UK), JNK, p-JNK, ERK, p-ERK, p38, p-p38 (Cell Signaling, Danvers, MA, USA), Col-1A, and GAPDH (Millipore, Billerica, MA, USA) antibodies were obtained.

### 2.3 Immunoblotting

Skin dermal fibroblasts cultured in 6-well plates were serum-starved for 16 h, pre-treated with an inhibitor (10 μM LY2109761, 10 μM SIS3, 10 μM EDHS-206, 10 μM SP600125, 30 μM PD98059, or 10 μM SB203580) for 30 min, and then treated with 10 ng/mL TGF-β1 for 24 h at 37°C. si-RNA for Sar1a was transfected into the HTSF for 24 h before serum starvation. The cells were lysed in RIPA buffer and then cleared lysate proteins were analyzed by SDS-PAGE and immunoblotting.

### 2.4 Immunofluorescence

Cells grown on slides were rinsed with phosphate-buffered saline (PBS), fixed with 4% paraformaldehyde for 15 min, and permeabilized in 0.2% Triton X-100 in PBS. Slides were incubated overnight at 4°C with the primary antibody. Slides were then incubated for 1 h with the appropriate fluorescence-labeled secondary antibody. All images were collected using an IX73 inverted fluorescence microscope (Olympus, Tokyo, JP). Fluorescence images were analyzed using ImageJ software (http://rsb.info.nih.gov/ij/).

### 2.5 Sar1a siRNA

The Sar1a siRNA was purchased from Bioneer Co. (Daejeon, South Korea). The control or target sequence of the Sar1a siRNA was human Sar1a. Sequences were as follows: Sense 5′-GAA CAG AUG CAA UCA GUG A-3′; Antisense 5′-CCA GUA UAU UGA CUG AUG U-3′. Poly ethylene glycol (PEG) conjugates, siRNA-s-s-PEG (siRNA-PEG) were prepared as described previously [10]. Briefly, the 3′-end hexylamine-modified siRNA was activated with the disulfide cross-linker, *N*-succinimidyl-3-(2-pyridyldithio) propionate and then coupled with PEG-SH to produce siRNA-PEG. To prepare siRNA polyelectrolyte complex micelles, the siRNA-PEG conjugate was simply mixed with polyethylenimine at a N/P ratio of 16 and incubated at room temperature for 15 min.

### 2.6 ELISA of PC-I

The cells were cultured until near confluence was reached, and then starved for 24 h in serum-free DMEM. After the experimental treatment was performed, the supernatants were collected from the cell cultures. PC type I α1/Col-1A secretion in the culture supernatants of NF or HTSF was determined via ELISA by using commercially available kits according to the manufacturer’s instructions (DuoSet kit, R&D system, Minneapolis, MN, USA).

### 2.7 Statistical analysis

Statistical significance between experimental groups was determined with student *t*-test or two-way analysis of variance with Sidak multiple comparisons test (Prism software, v7.0d; GraphPad Software, La Jolla, CA).

## 3 Results

### 3.1 Sar1a expression differs between NF and HTSF isolated from burn scar tissues

Studies have reported that various ECM proteins are overexpressed in HTSF and secreted from the cell, leading to abnormal wound healing or tissue remodeling [4,25]. It is hypothesized that the protein secretion system must be activated for this; however, only a few studies have investigated this. To verify this hypothesis, we used immunoblotting to investigate changes in the expression level of COPII-coat proteins.

**Figure 1:**
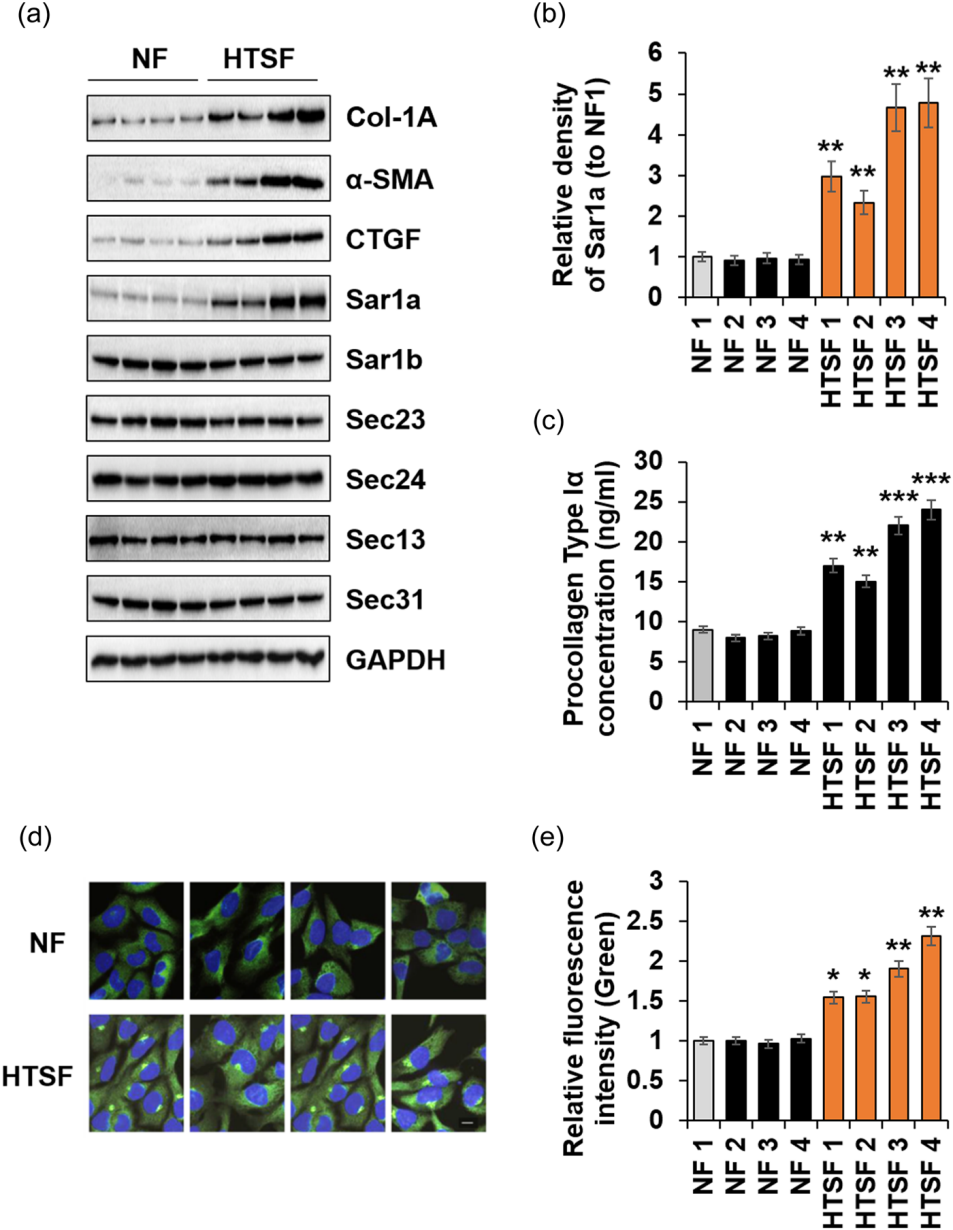
Differential expression of Sar1a between NF and HTSF. Isolated NFs or HTSFs were cultured in 6-well plates. The cell culture supernatant was collected for PC-I ELISA (c), and cell lysates were immunoblotted with antibodies against the indicated proteins (a). (a) Immunoblots of Col-1A, α-SMA, CTGF, Sar1a, Sar1b, Sec23, Sec24, Sec13, Sec31, Sec12p, and the loading control GAPDH. (b) Fold change of Sar1a was calculated (*n* = 3, ±SD); ** *p* < 0.01 vs NF 1. (c) NF or HTSF were cultured until near confluence, and they were then starved in serum-free DMEM for 24 h. Secreted PC-I was determined via ELISA (*n* = 3, ±SD); ** *p* < 0.01 and *** *p* < 0.001 vs NF 1. (d) Cultured cells were stained with anti-Sar1a (FITC, green) and DAPI (blue), and images were obtained using a fluorescence microscope (Scale bar: 10 μm). (e) Images were captured, and the fluorescence intensity was quantitated using ImageJ software (*n* = 3, ±SD); * *p* < 0.05 and **p < 0.01 vs NF 1.

HTSF markers, Col-1A, α-SMA, and CTGF genes were highly expressed in HTSF isolated from burn patients than in NF (Figure 1a and b), consistent with previous results [10]. Interestingly, Sar1a expression levels were significantly higher in HTSF than in NF; however, the expression levels of other COPII-coat proteins did not change significantly (Figure 1a and b). The expression level of Sar1a showed a similar trend to that of secreted PC-I in HTSFs (Figure 1b and c). Additionally, immunocytochemistry revealed a high Sar1a expression in HTSF (Figure 1d and e). In contrast to NF, the expression of Sar1a in HTSF was generally high, especially in the ER region. These results suggested that Sar1a upregulation was associated with increased PC-I secretion as part of HTSF hallmarks.

### 3.2 Sar1a is induced by TGF-β1

**Figure 2:**
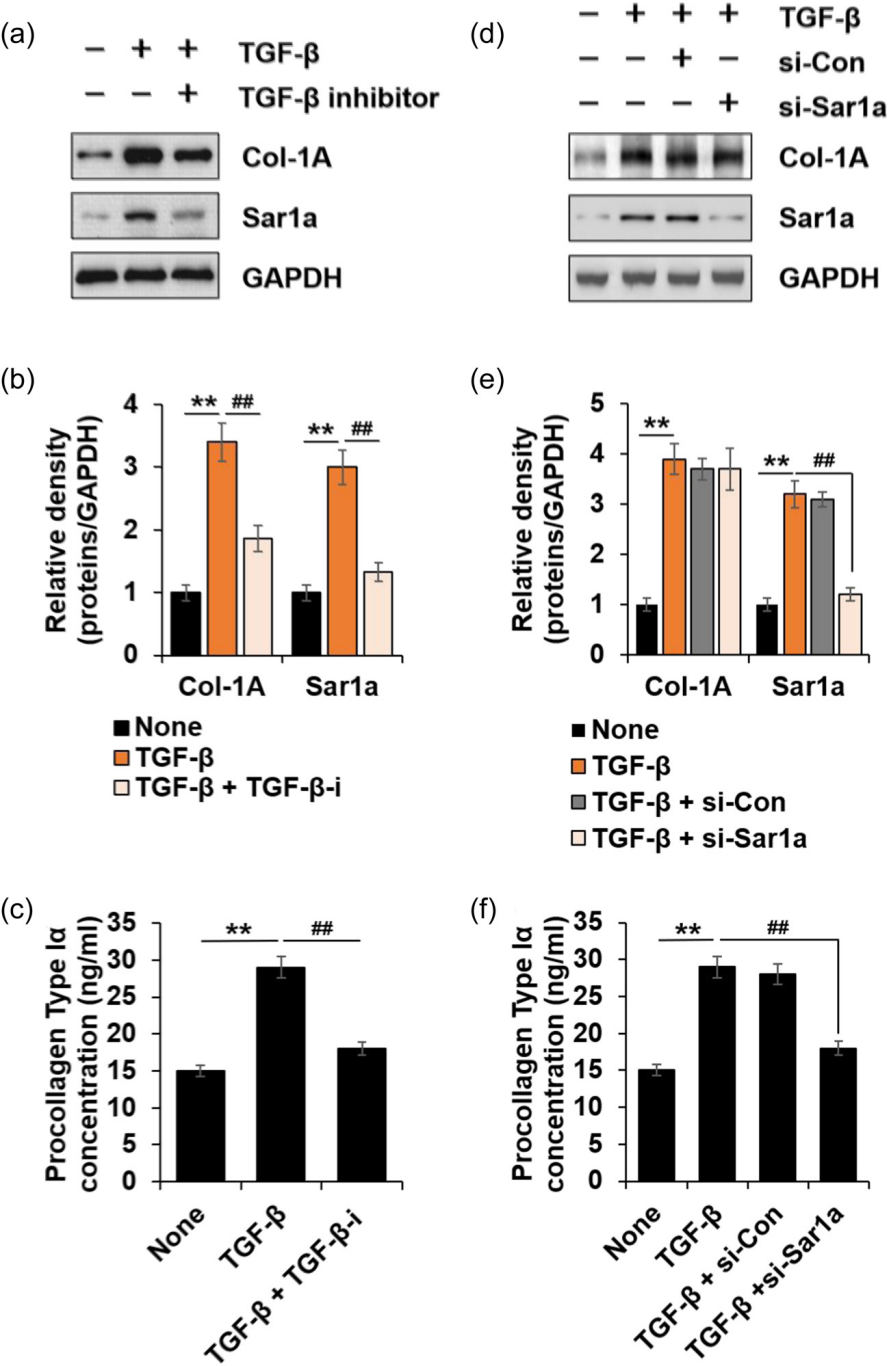
TGF-β1 stimulates induction of Sar1a. HTSF and Sar1a siRNA- or a control siRNA (si-con)-transfected HTSF were cultured in 6-well plates, serum-starved for 16 h, and then treated with or without 10 ng/mL TGF-β1 or TGF-β1 + 10 μM TGF-β inhibitor (LY2109761) for 24 h. The cell culture supernatant was collected for PC-I ELISA (c and f), and cell lysates were immunoblotted with antibodies against the indicated proteins (a and d). (a) Immunoblots of Col-1A, Sar1a, and the loading control GAPDH. (b) Fold change of Col-1A or Sar1a was calculated (*n* = 3, ±SD). None vs TGF-β1; ** *p* < 0.01. TGF-β1 vs TGF-β1 + TGF-β-i; ^##^ *p* < 0.01. (c) Secreted PC-I was determined via ELISA (*n* = 3, ±SD). None vs TGF-β; ** *p* < 0.01. TGF-β1 vs TGF-β1 + TGF-β-i; ^##^ *p* < 0.01. (d) Immunoblots of Col-1A, Sar1a, and the loading control GAPDH. (e) Fold change of Col-1A or Sar1a was calculated (*n* = 3, ±SD). None vs TGF-β1; ** *p* < 0.01. TGF-β1 vs TGF-β1 + TGF-β-i; ^##^ *p* < 0.01. (f) Secreted PC-I was determined via ELISA (*n* = 3, ±SD). None vs TGF-β; ** *p* < 0.01. TGF-β1 vs TGF-β1 + si-Sar1a; ^##^ *p* < 0.01.

TGF-β1 was upregulated in HTSF [26], which promoted the proliferation, Col formation, and differentiation of dermal fibroblasts through the intracellular Smad pathway [27]. We hypothesized that the altered expression of Sar1a in HTSF cells can be regulated via TGF-β1. To test this, we examined Sar1a expression in the presence of TGF-β1. HTSFs were treated with TGF-β1, and Sar1a expression level was detected via immunoblotting. The results revealed that Sar1a was induced in HTSF using TGF-β1 (Figure 2a and b). We also tested whether this effect was a receptor-dependent response with TGF-β1. We used LY2109761, a TGF-β receptor I kinase inhibitor, and we observed that LY2109761 not only inhibited Col-1A expression and PC-I secretion in the presence of TGF-β1 but also significantly inhibited Sar1a expression levels in HTSFs (Figure 2a–c). Additionally, we examined the effects of Sar1a depletion on Col-1A expression and PC-I secretion using a specific si-RNA for Sar1a. We observed no change in the expression of Col-1A induced by TGF-β1 in Sar1a-depleted cells (Figure 2d and e); however, the secretion of PC-I increased by TGF-β1 stimulation significantly decreased (Figure 2f). These results suggested that TGF-β1 signaling requires Sar1a expression in HTSF and upregulated Sar1a promotes PC-I secretion.

### 3.3 TGF-β1 induces Sar1a expression via TAK1 signaling

TGF-β induces multiple intracellular signaling pathways. Among them, the most important one is the Smad-dependent pathway through the Smad family of proteins, and it is a target for HTS treatment [27,28]. Smad-independent pathways via TAK1, RhoGTPase, and phosphatidylinositol-4,5-bisphosphate 3-kinase have also been extensively studied, and they have been reported to be involved in fibrotic disorders [29]. TAK1 is particularly involved in production of ECM and pathogenesis of fibrosis [29]. TGF-β-induced fibronectin expression is mediated by TAK1 through MKK4-JNK signaling cascade in fibroblasts [30], and TAK1-deficient fibroblasts exhibit a decreased profibrotic response to TGF-β1 stimulation [31].

**Figure 3:**
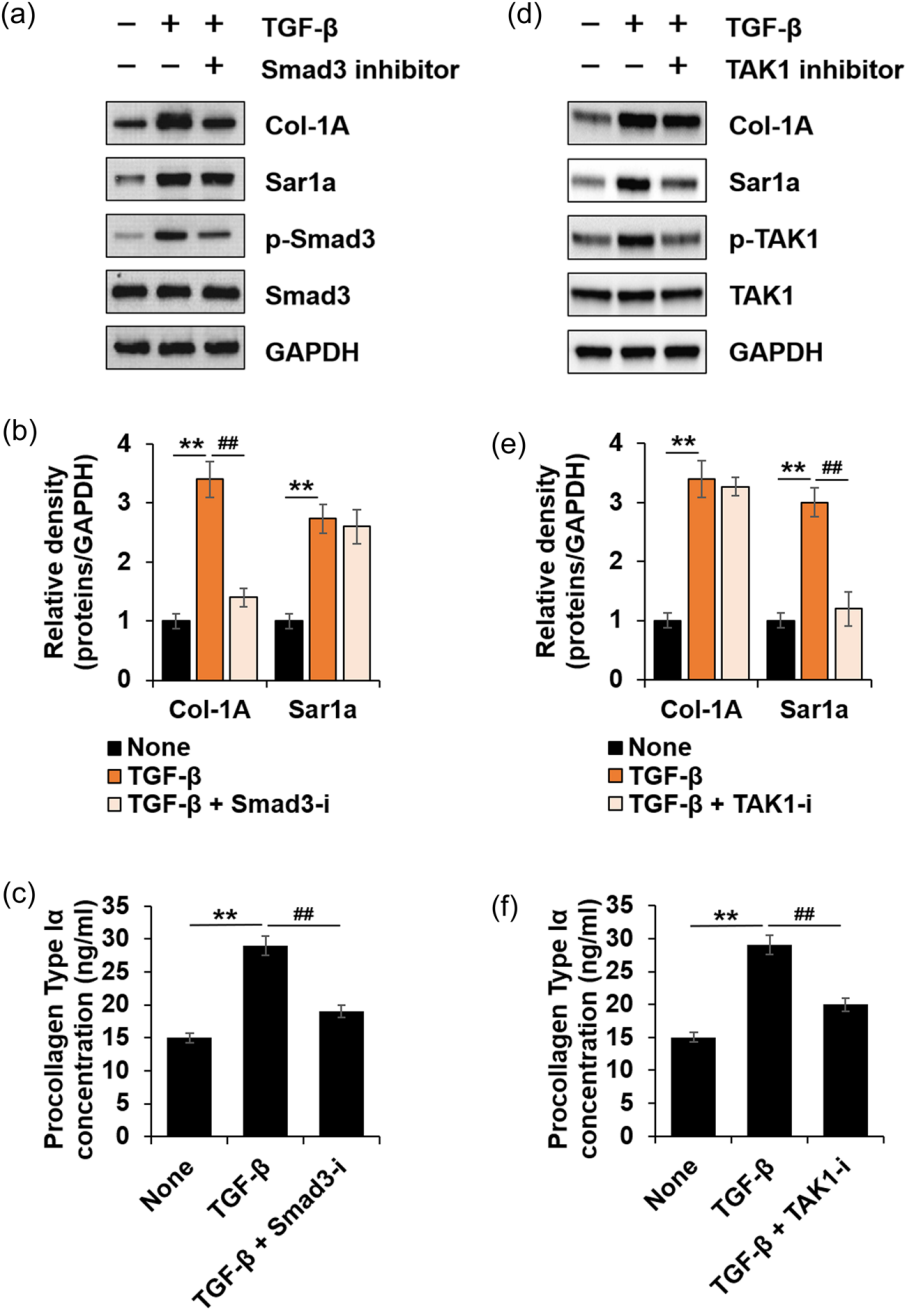
TAK1 is involved in TGF-β1-induced Sar1a expression. HTSF cultured in 6-well plates were serum-starved for 16 h, and they were then treated with or without 10 ng/mL TGF-β1, TGF-β1 + 10 μM SIS3, or TGF-β1 + 10 μM TAK1 inhibitor (EDHS-206) for 24 h. The cell culture supernatant was collected for PC-I ELISA (c and f), and cell lysates were immunoblotted with antibodies against the indicated proteins (a and d). (a) Immunoblots of Col-1A, Sar1a, phosphor-Smad3, Smad3, and GAPDH. (b) Fold change of Col-1A or Sar1a was calculated (*n* = 3, ±SD). None vs TGF-β1, ** *p* < 0.01. TGF-β1 vs TGF-β1 + Smad3-I; ^##^ *p* < 0.01. (c) Secreted PC-I was determined via ELISA (*n* = 3, ±SD). None vs TGF-β1; ** *p* < 0.01. TGF-β1 vs TGF-β1 + Smad3-I; ^##^ *p* < 0.01. (d) Immunoblots of Col-1A, Sar1a, phosphor-TAK1, TAK1, and GAPDH as the loading control. (e) Fold change of Col-1A or Sar1a was calculated (*n* = 3, ±SD). None vs TGF-β1; ** *p* < 0.01. TGF-β1 vs TGF-β1 + TAK1-I; ^##^ *p* < 0.01. (f) Secreted PC-I was determined via ELISA (*n* = 3, ±SD). None vs TGF-β1; ** *p* < 0.01. TGF-β1 vs TGF-β1 + TAK1-i; ^##^ *p* < 0.01.

To determine the signaling mechanisms for Sar1a expression induced by TGF-β1, we used specific inhibitors to investigate the signaling molecules required for TGF-β1-induced Sar1a expression, SIS3, and EDHS-206 (selective TAK1 inhibitor). Smad3 inhibitors display significant negative effects on TGF-β1-induced Col-1A expression or PC-I secretion but not on Sar1a expression in HTSF (Figure 3a–c). While TAK1 inhibitor did not significantly affect Col-1A expression, it significantly inhibited Sar1a expression or PC-I secretion (Figure 3d–f). These results suggested that TGF-β1 induced Sar1a expression via the TAK1 signaling pathway and it is involved in PC-I secretion.

### 3.4 JNK and p38 participate in TGF-β1-induced Sar1a expression

JNK and p38 are downstream targets of TAK1 activation in tissue injury response and fibrosis [30]. ERK and JNK inhibitors significantly suppressed CTGF-induced expression of α-SMA and Col-1A in HTSF of rabbit ear model [32].

**Figure 4:**
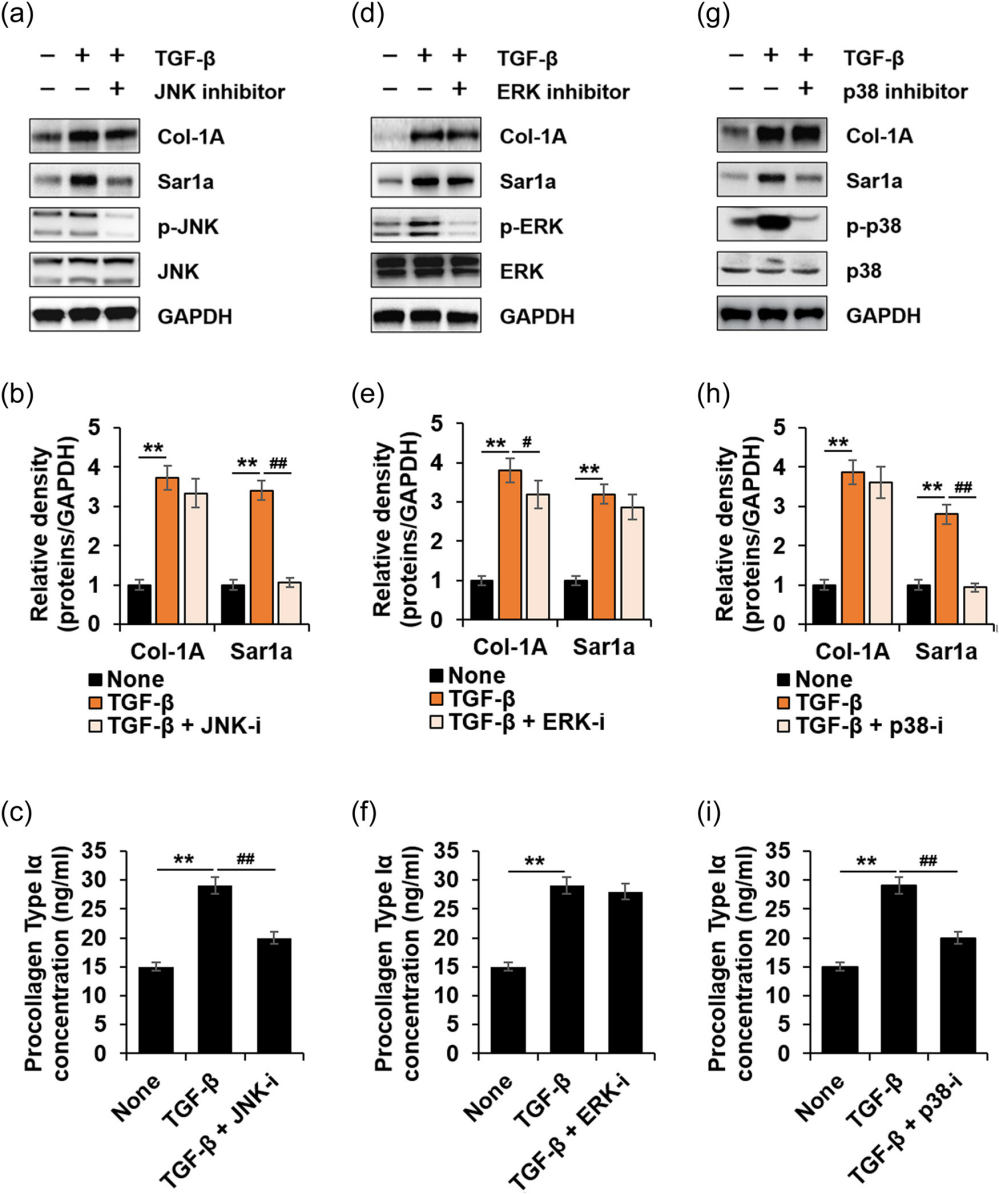
JNK and p38 are involved in TGF-β1-induced Sar1a expression. HTSF were cultured in 6-well plates, serum-starved for 16 h, and then treated with or without 10 ng/mL TGF-β1, TGF-β1 + 10 μM JNK inhibitor (SP600125), 50 μM ERK inhibitor (PD98059), or TGF-β1 + 10 μM p38 inhibitor (SB203580) for 24 h. The cell culture supernatant was collected for PC-I ELISA (c, f, and i), and cell lysates were immunoblotted with antibodies against the indicated proteins (a, d, and g). (a) Immunoblots of Col-1A, Sar1a, phosphor-JNK, JNK, and GAPDH as the loading control. (b) Fold change of Col-1A or Sar1a was calculated (*n* = 3, ±SD). None vs TGF-β1; ** *p* < 0.01. TGF-β1 vs TGF-β1 + JNK-i; ^##^ *p* < 0.01. (c) Secreted PC-I was determined via ELISA (*n* = 3, ±SD). None vs TGF-β1; ** *p* < 0.01. TGF-β1 vs TGF-β1 + JNK-i; ^##^ *p* < 0.01. (d) Immunoblots of Col-1A, Sar1a, phosphor-ERK, ERK, and GAPDH as the loading control. (e) Fold change of Col-1A or Sar1a was calculated (*n* = 3, ±SD). None vs TGF-β1; ** *p* < 0.01. TGF-β1 vs TGF-β1 + ERK-I; ^#^ *p* < 0.05. (f) Secreted PC-I was determined via ELISA (*n* = 3, ±SD). None vs TGF-β1; ** *p* < 0.01. (g) Immunoblots of Col-1A, Sar1a, phosphor-p38, p38, and GAPDH as the loading control. (h) Fold change of Col-1A or Sar1a was calculated (*n* = 3, ±SD). None vs TGF-β1; ** *p* < 0.01. TGF-β1 vs TGF-β1 + p38-I; ^##^ *p* < 0.01. (i) Secreted PC-I was determined via ELISA (*n* = 3, ±SD). None vs TGF-β1; ** *p* < 0.01. TGF-β1 vs TGF-β1 + p38-i; ^##^ *p* < 0.01.

Next we investigated whether MAPKs (JNK, ERK, and p38) are involved in TGF-β1-induced Sar1a expression in HTSFs. The expression level of Sar1a decreased following treatment with JNK or p38 inhibitor (Figure 4a,b,g, and h) while it did not significantly change with ERK inhibitor in HTSF (Figure 4d and e). Additionally, JNK or p38 inhibitor interrupted TGF-β1-induced PC-I secretion but not Col-1A expression (Figure 4a–c and g–i), while ERK inhibitor slightly decreased Col-1A expression but had not significant effect on PC-I secretion (Figure 4a–i). These results indicated that Sar1a upregulation is a positive regulator for PC-I secretion via the TGF-β1-mediated TAK1-JNK and TAK1-p38 pathways in HTSF.

## 4 Discussion

Development of HTS involves a complex interplay between cells and cytokines as well as local and systemic factors exerted on dermal fibroblasts, resulting in a distinctive HTSF phenotype [33]. Particularly, TGF-β is the most important key to regulate the expression of genes such as Col-1, -3, fibronectin, α-SMA, and CTGF, which are major markers of HTSF [34]. In this study, we observed that the expression of Sar1a as well as other HTSF marker genes was higher in HTSF than in NF cells (Figure 1). As Sar1 is responsible for COPII vesicle trafficking, altered expression of Sar1a in HTSF may enable rapid transport of PC out of the cell. Our data showed that TGF-β1-induced Col-1A expression was not affected in sar1a-deficient HTSF; however, PC-I secretion was impaired (Figure 2).

Many studies have shown that PC secretion is dependent on COPII; however, the mechanism remains unclear and controversial. A key question is whether COPII vesicles are flexible enough to accommodate large cargoes as they are approximately 60–90 nm in size and PC-I bundle is 300 nm [35]. It has been reported that deficiency of Sar1a and Sar1b inhibits the export of PC-I [36], and local concentration of Sar1-GTP determines the timing of membrane cleavage and COPII vesicle size [37]. Further, COPII proteins and GTPase activity of Sar1 form large COPII-coated membrane vesicles that transport PC-I out of the ER [38]. Concomitantly, our findings indicate that alteration of HTSF upregulated Sar1a protein, thereby promoting PC-I secretion. Recently, new PC-I secretion models using super-resolution light microscopy have been proposed [39], but no definitive conclusions have been reached. It is likely that new developments in super-resolution light microscopy and correlative light-electron microscopy will drive new understanding here.

We demonstrated that TGF-β1 induced Sar1a and Col-1A expression, which were TGF-β receptor-dependent (Figure 2). These findings suggested a novel role of TGF-β1 in HTSF. Next we investigated the cellular mechanism from TGF-β1 to Sar1a expression and PC-I secretion. The Smad3 inhibitor effectively decreased TGF-β1-induced Col-1A expression in HTSF but had no effect on Sar1a expression (Figure 3a and b) whereas TAK1 inhibitor significantly inhibited Sar1a expression but had no effect on Col-1A expression (Figure 3d and e). However, both inhibitors decreased PC-I secretion. Furthermore, we showed that both JNK and p38 participated in TGF-β1-induced Sar1a expression, but ERK did not (Figure 4). Taken together, these results suggested that TGF-β1 induced Sar1a expression through TAK1 signaling and excessive PC-1 secretion required both Smad3- and TAK1-mediated signaling in HTSF.

Contrary to our results (Figure 4a and b), it was reported that CTGF-induced JNK activation was involved in Col-1A expression in HTSF of a rabbit model [32]. This difference may be due to differences in the pathogenic processes of HTS between humans and animals [40], as well as other stimuli used for Col-1A expression. However, study using cDNA microarrays suggested that the JNK pathway did not affect the TGF-β-induced transcriptional activity of Col-1A in human dermal fibroblasts [41]. Even activated by cytokines such as TNF-α or pharmacological molecules such as 5-fluoro-uracil, JNK induces c-Jun phosphorylation, interfering with Smad3-dependent transcription via the TGF-β/Smad3 signaling pathway [42–44].

There are microRNA (miR) studies targeting Sar1a. One is a study that miR-34c suppresses the expression of Sar1a to reduce proinsulin secretion [45], and the other is a study that miR-34 family members in early- to mid-gestational fetal keratinocytes contribute to scarless wound healing by targeting the TGF-β pathway [46]. In the latter study, the authors seem to confuse Sar1a with Smad anchor for receptor activation, but the miR-34 family could be considered a very promising candidate for HTS research and therapy. Their study showed that TGF-β receptor-I, -II, Smad3, and Smad4 as well as Sar1a are potential target genes of the miR-34 family. However, it is necessary to study whether the miR-34 family members could suppress the expression of target genes and HTS marker genes in HTSF.

In conclusion, we proposed that Sar1a expression was high in HTSF cells, which efficiently modulated COPII vesicle traffic, leading to an increased PC-I secretion via TGF-β1-mediated signaling. These insights may aid the development of novel anti-scar or fibrosis drugs.
